# Supplementary material for: Serum cystatin-C and all-cause mortality in patients with hypertrophic cardiomyopathy: a retrospective cohort study
Source: PeerJ. 2025 Nov 7;13:e19631. doi: 10.7717/peerj.19631 (PMC12599373; doi:10.7717/peerj.19631)
Supplement: Supplemental Information 3 [file peerj-13-19631-s003.docx]

Supplementary Table 1. Univariable Cox’s regression analysis for all-cause mortality in the whole cohort

| Variables | Change | Univariable | | |
| --- | --- | --- | --- | --- |
|  |  | HR | 95 CI % | p |
| Age (year) | Per 1-year increase | 1.03 | 1.02-1.05 | <0.001 |
| COPD | Yes vs. no | 3.75 | 2.15-6.54 | <0.001 |
| Prior TE | Yes vs. no | 2.07 | 1.00-4.27 | 0.050 |
| NYHA III/IV | Yes vs. no | 3.05 | 2.01-4.63 | <0.001 |
| AF | Yes vs. no | 2.26 | 1.45-3.52 | <0.001 |
| Warfarin | Yes vs. no | 2.10 | 1.19-3.72 | 0.011 |
| Diuretic | Yes vs. no | 2.97 | 1.94-4.55 | <0.001 |
| Beta-blocker | Yes vs. no | 0.62 | 0.41-0.95 | 0.029 |
| BUN (mmol/L) | Per 1 mmol/L increase | 1.05 | 1.02-1.07 | <0.001 |
| eGFR (ml/min*1.73m^2^) | Per 1 ml/min*1.73m^2^ increase | 0.985 | 0.976-0.993 | 0.001 |
| Creatinine (umol/L) | Per 1 umol/L increase | 1.001 | 1.000-1.003 | 0.086 |
| Cystatin-C (mg/L) | Per 1 mg/L increase | 1.36 | 1.19-1.57 | <0.001 |
| Uric acid (umol/L) | Per 1 umol/L increase | 1.003 | 1.001-1.005 | 0.001 |
| Triglyceride (mmol/L) | Per 1 mmol/L increase | 0.64 | 0.47-0.88 | 0.005 |
| Total cholesterol (mmol/L) | Per 1 mmol/L increase | 0.70 | 0.56-0.87 | 0.001 |
| LDL-C (mmol/L) | Per 1 mmol/L increase | 0.65 | 0.49-0.86 | 0.002 |
| LVEDD (mm) | Per 1 mm increase | 0.96 | 0.92-1.00 | 0.028 |
| LA (mm) | Per 1 mm increase | 1.03 | 1.01-1.06 | 0.018 |
| LVPW (mm) | Per 1 mm increase | 1.10 | 1.04-1.15 | 0.001 |
| EF (%) | Per 1 percent increase | 0.97 | 0.95-0.99 | 0.005 |

Only variables which were significantly associated with all-cause mortality (p < 0.100) were shown.

AF: atrial fibrillation; BUN: blood urea nitrogen; CI: confidence interval; COPD: chronic obstructive pulmonary disease; EF: ejection fraction; eGFR: estimated glomerular filtration rate; HCM: hypertrophic cardiomyopathy; HR: hazard ratio; LA: left atria diameter; LDL-C: low density lipoprotein cholesterol; LVEDD: left ventricular end-diastolic dimension; LVOTO: left ventricular outflow track obstruction; LVPW: left ventricular posterior wall; NYHA: New York Heart Association; SCD: sudden cardiac death; TE: thromboembolism event

Figure S1. Time-dependent AUCs of eGFR (MDRD formula) for predicting all-cause mortality with the extension of time


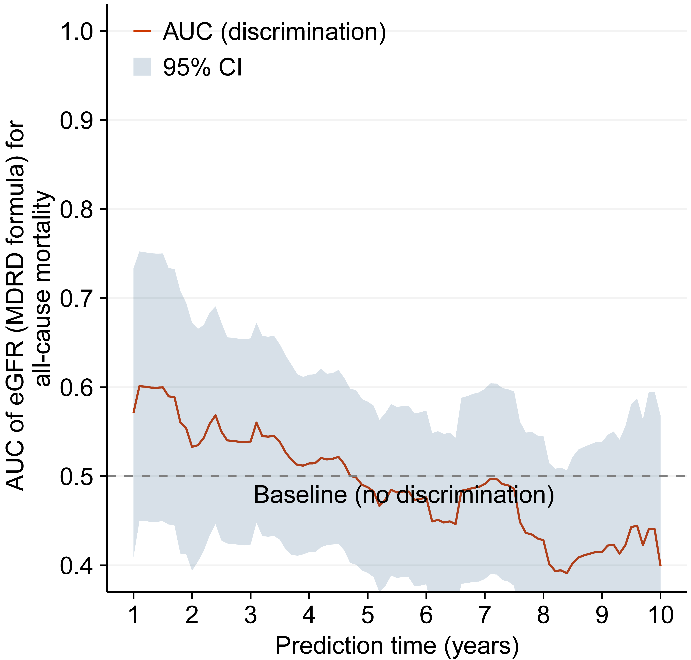


Notes: Time-dependent AUC at each time of the follow-up was plotted to evaluate the accuracy of eGFR (MDRD formula) in the prediction of all-cause mortality. A generally accepted approach suggests that an AUC of less than 0.60 reflects poor discrimination; 0.60 to 0.75, possibly helpful discrimination; and more than 0.75, clearly useful discrimination. eGFR (MDRD) shows poorer discrimination with time extending. AUC, area under the curves; CI, confidence interval; eGFR, estimated glomerular filtration rate; HCM, hypertrophic cardiomyopathy

Figure S2. Time-dependent AUCs of eGFR (CKD-EPI formula) for predicting all-cause mortality with the extension of time.


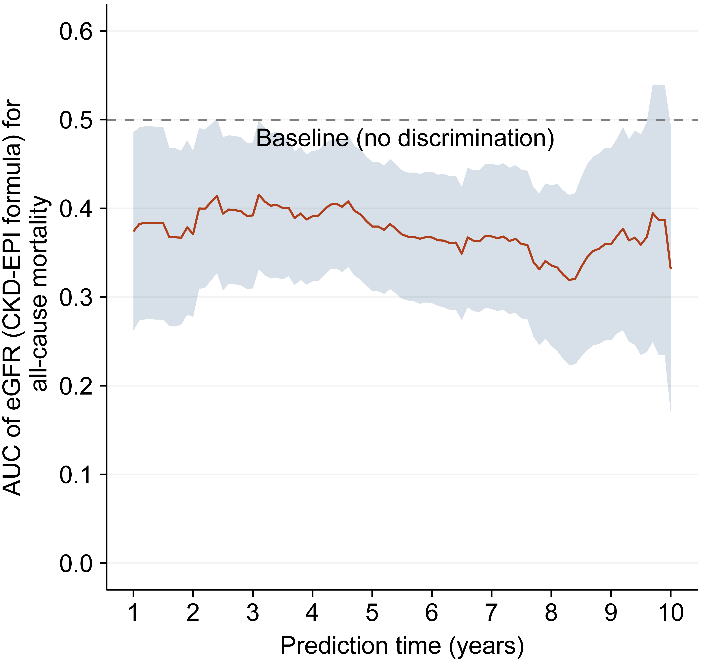


Notes: Time-dependent AUC at each time of the follow-up was plotted to evaluate the accuracy of eGFR (CKD-EPI formula) in the prediction of all-cause mortality. A generally accepted approach suggests that an AUC of less than 0.60 reflects poor discrimination; 0.60 to 0.75, possibly helpful discrimination; and more than 0.75, clearly useful discrimination. AUCs of eGFR (CKD-EPI) at most time points are below 0.4, showing poor discrimination. AUC, area under the curves; CI, confidence interval; eGFR, estimated glomerular filtration rate; HCM, hypertrophic cardiomyopathy

Figure S3. Time-dependent AUCs of serum creatinine for predicting all-cause mortality with the extension of time


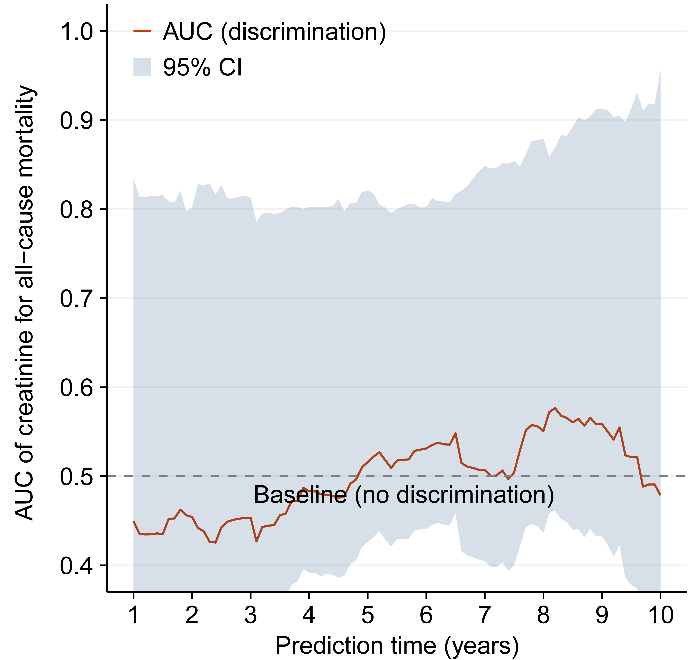


Notes: Time-dependent AUC at each time of the follow-up was plotted to evaluate the accuracy of serum creatinine in the prediction of all-cause mortality. A generally accepted approach suggests that an AUC of less than 0.60 reflects poor discrimination; 0.60 to 0.75, possibly helpful discrimination; and more than 0.75, clearly useful discrimination. Serum creatinine shows poor discrimination across the whole follow-up. AUC, area under the curves; CI, confidence interval; HCM, hypertrophic cardiomyopathy.

Figure S4. Pearson’s correlation coefficients between pairs of independent variables.


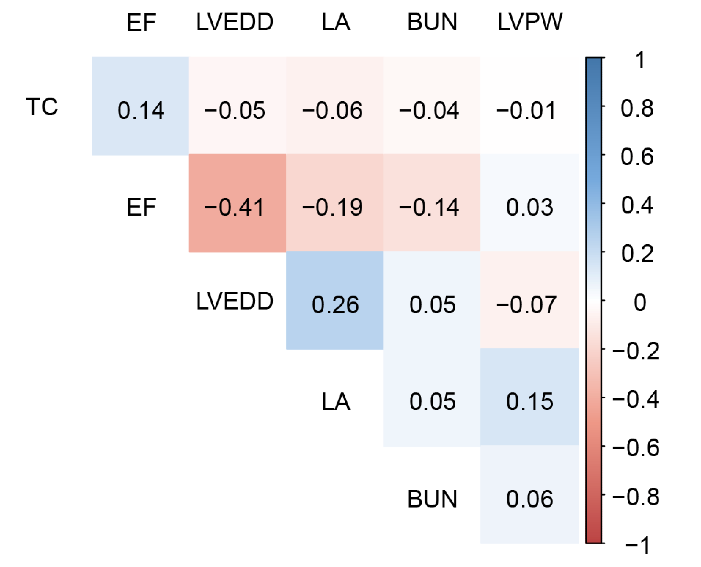


Notes: VIF values and Pearson’s correlation coefficients were used to evaluate the degree of multicollinearity among the independent variables. All the correlation coefficients between pairs of independent variables were < 0.7 and the VIF values were close to 1, indicating no collinearity among the independent variables. BUN, blood urea nitrogen; LA, left atrium; LVEDD, left ventricular end diastolic diameter; LVPW, left ventricular posterior wall; EF, ejection fraction; VIF, variance inflation factor.

Figure S5. Time-dependent AUCs of serum cystatin-C for predicting all-cause mortality with the extension of time in the HCM patients with eGFR ≥ 60 mL/min/1.73 m^2^


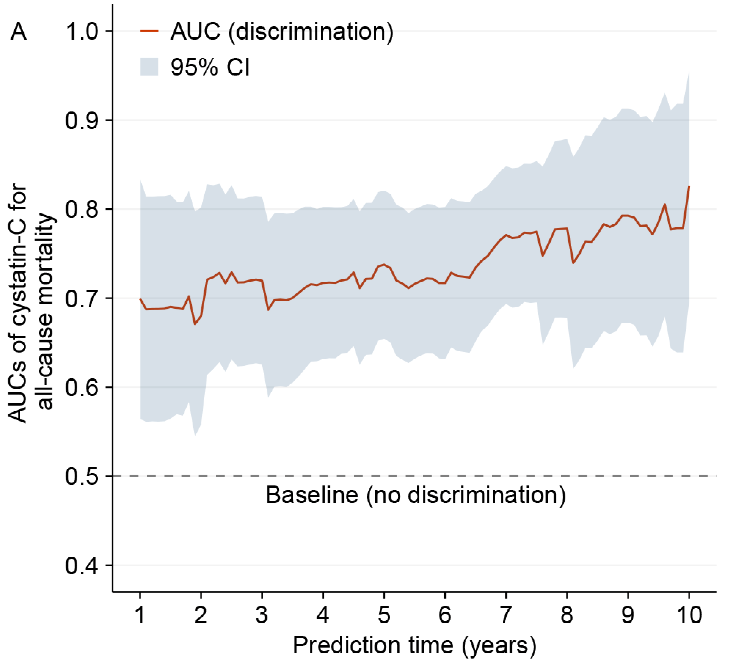


Notes: Time-dependent AUC at each time of the follow-up was plotted to evaluate the accuracy of serum cystatin-C in the prediction of all-cause mortality in the HCM patients with eGFR ≥ 60 mL/min/1.73 m^2^. A generally accepted approach suggests that an AUC of less than 0.60 reflects poor discrimination; 0.60 to 0.75, possibly helpful discrimination; and more than 0.75, clearly useful discrimination. AUC, area under the curves; CI, confidence interval; HCM, hypertrophic cardiomyopathy.
